# Urea application promotes amino acid metabolism and membrane lipid peroxidation in *Azolla*

**DOI:** 10.1371/journal.pone.0185230

**Published:** 2017-09-25

**Authors:** Jiana Chen, Min Huang, Fangbo Cao, P. Pardha-Saradhi, Yingbin Zou

**Affiliations:** 1 Southern Regional Collaborative Innovation Center for Grain and Oil Crops (CICGO), Hunan Agricultural University, Changsha, China; 2 Department of Environmental Studies, University of Delhi, Delhi, India; Stellenbosch University, SOUTH AFRICA

## Abstract

A pot experiment was conducted to evaluate the effect of urea on nitrogen metabolism and membrane lipid peroxidation in *Azolla pinnata*. Compared to controls, the application of urea to *A*. *pinnata* resulted in a 44% decrease in nitrogenase activity, no significant change in glutamine synthetase activity, 660% higher glutamic-pyruvic transaminase, 39% increase in free amino acid levels, 22% increase in malondialdehyde levels, 21% increase in Na^+^/K^+^- levels, 16% increase in Ca^2+^/Mg^2+^-ATPase levels, and 11% decrease in superoxide dismutase activity. In terms of H_2_O_2_ detoxifying enzymes, peroxidase activity did not change and catalase activity increased by 64% in urea-treated *A*. *pinnata*. These findings suggest that urea application promotes amino acid metabolism and membrane lipid peroxidation in *A*. *pinnata*.

## Introduction

*Azolla* is a floating aquatic fern that is widely distributed throughout temperate and tropical freshwater ecosystems. It establishes symbiotic associations with nitrogen (N)-fixing blue-green algae, and this association is mainly responsible for its high productivity and its ability to fix N at high rates [1]. For centuries, *Azolla* has been employed as green manure in rice in China and Vietnam and more recently in other Asian and African countries [2]. The basal application of *Azolla* at 10–12 t∙ha^–1^ increases soil N by 50–60 kg∙ha^–1^ and reduces the requirement for chemical N fertilizers in rice production by 30–35 kg∙ha^–1^ [3]. Besides its utilization as a green manure, *Azolla* can be used as an animal feed, a human food, a medicine, and a water purifier. It may also be used for the production of hydrogen fuel, the production of biogas, the control of weeds, the control of mosquitoes, and the reduction of ammonia volatilization which accompanies the application of chemical N fertilizer [4].

**Fig 1:**
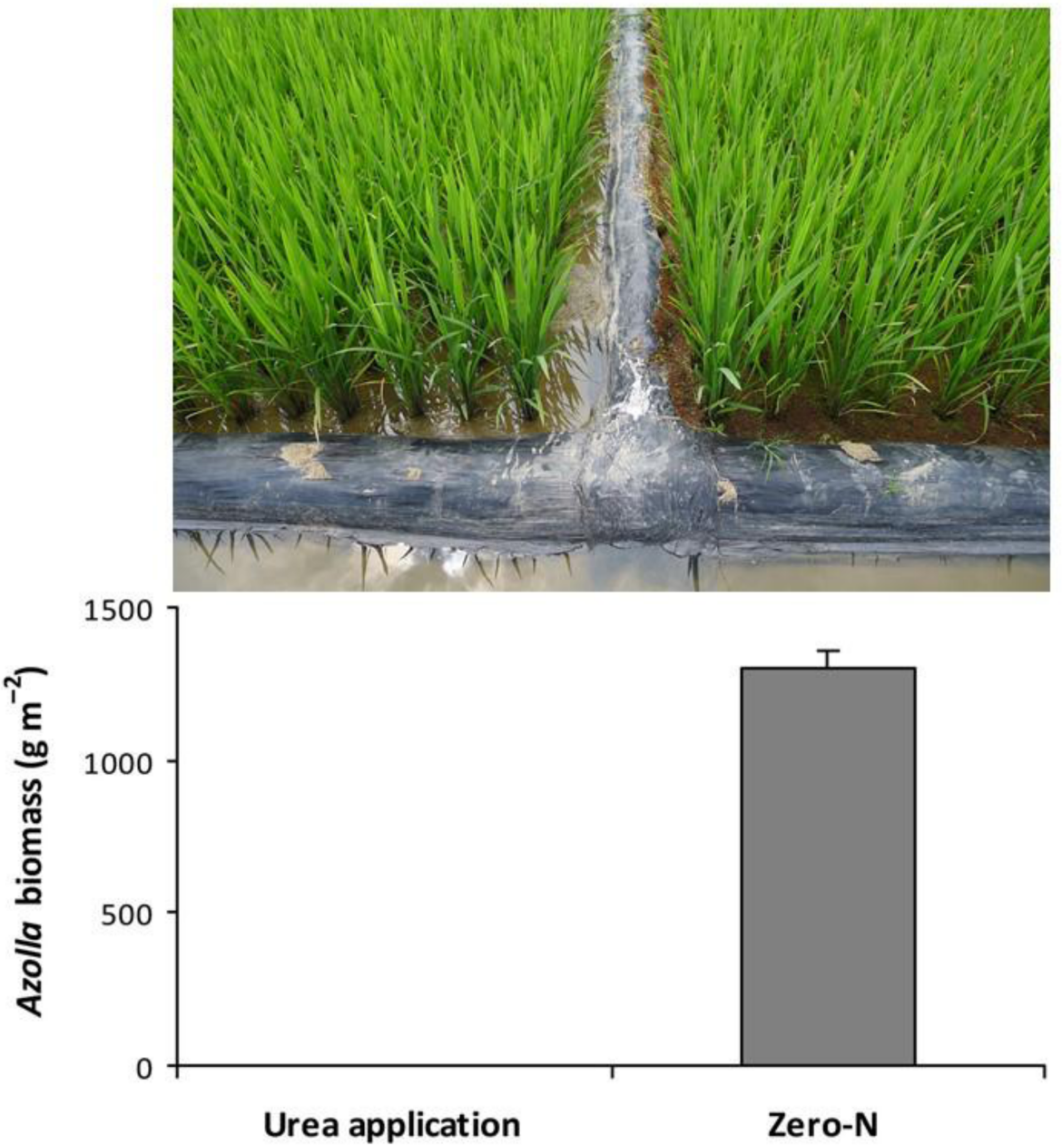
Effect of urea application on the growth of *Azolla*. The picture and data were obtained from a long-term field experiment with double rice cropping from 2008 onwards in Liuyang County, Hunan Province, China in early rice-growing season in 2016. See Qin et al. [7] for the details of the experiment. Vertical bars represent SE (*n* = 4).

The growth of *Azolla* is influenced by various environmental factors, and it is well documented that *Azolla* prefers sites that are rich in all essential plant nutrients, except for N [5, 6]. Similarly, in 2016, we observed a considerable amount of *A*. *pinnata* in zero-N plots but not in urea-applied plots previously used in a long-term field experiment with double rice cropping that was initiated in 2008 (Fig 1). This suggests that urea is a stressor to *Azolla* and a decline in Azolla biodiversity in rice field ecosystems. Furthermore, urea application synchronously inhibits the growth of *Azolla* and the amount of N fixed [2, 8], thereby indicating that there may be a link between the effect of urea application on the growth and N metabolism in *Azolla*. Currently, information on the effect of urea application on N metabolism in *Azolla* is limited.

Plants subjected to adverse environments usually undergo alterations in cell membrane structure and function [9, 10]. Membrane lipid peroxidation is a major contributor to these alterations, which presumably can lead to further deleterious consequences such as inhibition of ATPases [11, 12]. Malondialdehyde (MDA) is one of the major products of membrane lipid peroxidation and has been used as an indicator of membrane lipid peroxide levels [13, 14]. Additionally, in response to adverse conditions (e.g., dry, high, or low temperature, waterlogging), antioxidant enzymes such as superoxide dismutase (SOD), peroxidase (POD), and catalase (CAT) are produced to remove active oxygen radicals [15]. However, whether these physiological processes are induced by urea application in *Azolla* remain unclear.

In the present study, a pot experiment was conducted to determine several parameters associated with N metabolism and membrane lipid peroxidation in *Azolla* with and without urea application. The objective of this study was to determine the effect of urea application on the metabolism and membrane lipid peroxidation in *Azolla*.

## Materials and methods

### Ethics statements

No specific permissions were required for the activities conducted in this study. The experiments did not involve endangered or protected species.

### Plant culture

*A*. *pinnata* plants used in this study were collected from a zero-N plot used in a long-term field experiment with double rice cropping that was initiated in 2008 in Liuyang County, Hunan Province, China during the early rice-growing season of 2016 (Fig 1). Twelve plastic pots (22 cm × 16 cm × 7 cm; length × width × height) were filled with distilled water to a depth of 5 cm. The pots were separated into two groups: one group (*n* = 6) received urea (46% N) at a rate of 1.15 g∙pot^–1^ (equivalent to the recommended field rate of 150 kg N∙ha^–1^) and the other group (*n* = 6) did not receive N fertilizers (control). Uniformly sized *Azolla* plants were selected and cultured in the pots at a rate of 10 g fresh weight (FW)∙pot^–1^ under laboratory conditions.

### Data collection

Each pot was treated as one replicate. All plants in each pot were sampled at 48 h after culture. Approximately 0.1 g of fresh plant tissues was randomly collected and used in measuring nitrogenase activity. The rest of the plants were frozen in liquid N_2_ and stored at -80°C for the determination of glutamine synthetase (GS), glutamic-pyruvic transaminase (GPT), Na^+^/K^+^-ATPase, Ca^2+^/Mg^2+^-ATPase, SOD, POD, and CAT activity and MDA and free amino acid levels. The determination methods of various enzymes were as follows:

Nitrogenase activity was determined by an acetylene reduction assay [16] using a gas chromatograph. Fresh plant tissues (0.1 g) were placed in 20 ml bottles. Two layers of cheesecloth wetted with 0.2 ml of water were placed in each bottle to supply moisture. Bottles were capped with rubber serum stoppers, and reactions were incubated at 25°C for 30 min under 0.25 atm O_2_, 0.65 atm argon, and 0.1 atm acetylene. The reactions were initiated by the injection of acetylene and were terminated by adding 0.5 ml of 50% trichloroacetic acid with a syringe. Ethylene was assayed by the procedure utilized by Koch and Evans [17]. Appropriate control reactions were included.

GS activity was measured by a γ-glutamylhydroxamate assay [18], and one unit (U) of activity was defined as an absorbance change of 0.01 per min at a wavelength of 540 nm. One unit of GS activity is the amount of enzyme catalyzing the formation of 1 μmol γ-glutamylhydroxamate per min at 25°C.

GPT activity was determined by a modified method of Cabaud et al. [19], which was originally used for the determination of transaminase activity in serum samples. Approximately 0.1 g of a frozen sample was homogenized at 4°C in 0.05 M Tris-HCl extraction buffer (pH 7.2). The crude extract was centrifuged at 8,000*g* for 10 min. The enzyme activity in the supernatant was measured at 37°C in a reaction buffer containing 200 mM DL-alanine and 2 mM α-ketoglutarate. Around 30 min later, the reaction was stopped by adding 0.5 mL of 2, 4-dinitrophenylhydrazine. The absorbance of the reaction solution (pyruvic acid) was measured at a wavelength 505 nm. Enzyme activity was calculated from a standard calibration curve, and 1 U of the activity corresponded to the formation of 1 μmol pyruvic acid per 30 min.

Na^+^/K^+^- and Ca^2+^/Mg^2+^-ATPase activity was determined by measuring the amount of inorganic phosphate (Pi) released from ATP [20], and 1 U of the activity corresponded to 1 μmol Pi released per hour. The enzymatic reactions were performed using assay kits (Jiancheng Biological Engineering Research Institute, Nanjing, China).

SOD activity was determined by monitoring the rate of reduction of nitroblue tetrazolium (NBT) [21], and 1 U of enzyme activity was defined as the amount of enzyme causing a 50% inhibition in the NBT reduction rate. Frozen sample (0.5 g) were homogenized in an ice cold 100 mM EDTA–phosphate buffer (pH 7.8). The homogenate was centrifuged (10,000×g) for 20 min at 4°C and supernatant was used as the enzyme extract. The activity was determined by using the photochemical p–nitrobluetetrazolium chloride (NBT) reduction method.

POD activity was measured using the guaiacol oxidation method [22]. Frozen sample (0.1 g) was homogenised in 2 mL 50 mM phosphate buffer (pH 6.1). The homogenate was centrifuged at 10,000×g and the supernatant was used as enzyme extract. Peroxidaseactivity was determined with guaiacol as the substrate in a total volume of 3 mL. Increase in the absorbance due to oxidation of guaiacol (ε = 25.5 mM^−1^ cm^−1^) was measured at 470nm. 1 U of enzyme activity was defined as an absorbance change of 0.01 per min at a wavelength of 470 nm.

CAT activity was determined by monitoring the rate of decomposition of H_2_O_2_ [23]. Frozen sample (0.1 g) was homogenised with 3 mL 50 mM potassium phosphate buffer containing 1 mM EDTA (pH 7.0) and centrifuged at 6000×g for 15 min. The supernatant obtained was used as enzyme extract. The concentration of H_2_O_2_ in each sample was calculated using the extinction coefficient (ε = 39.4 mM^−1^ cm^−1^) and and one unit (U) of CAT activity is the amount of enzyme dissociating 1 nmol H_2_O_2_ min^−1^ at a wavelength of 240 nm.

The concentration of MDA was assayed by measuring the amount of thiobarbituric acid reactive substances [24]. Frozen sample (0.5 g) were crushed in 10 mL of 80:20 (v/v) ethanol and water solution, followed by centrifugation at 3000×g for 10 min. Absorbance was read at 440, 532 and 600 nm with the help of appropriate equations the TBARS (thiobarbituric acid reactive substances) as MDA equivalents were determined.

The concentration of free amino acids was determined by the ninhydrin colorimetric method [25]. Frozen sample (0.5 g) was crushed in 5 ml of 10% acetic acid and diluted to 100 ml with distilled water. Then the extract was filtered. The total free amino acids were determined by taking 1 ml of filtered liquid 3 ml of hydrogenated ninhydrin reagent in 25 ml volumetric flask. The mixture was heated for 15 min in boiling water bath and cooled to room temperature. It was then diluted to 25 ml with distilled water and laid aside for 10–15 min. The optical density of the solution was checked at 570 nm by spectrophotometer, using distilled water as a blank. Using leucine as a standard, standard curve was prepared by plotting the absorbance of a series of working standards against their respective concentrations.

### Statistical analysis

Data were analyzed by ANOVA (Statistix 8.0, Analytical Software, Tallahassee, FL, USA). The statistical model used included sources of variations due to replication and treatment.

## Results and discussion

### Effect of urea application on N metabolism in *Azolla*

**Fig 2:**
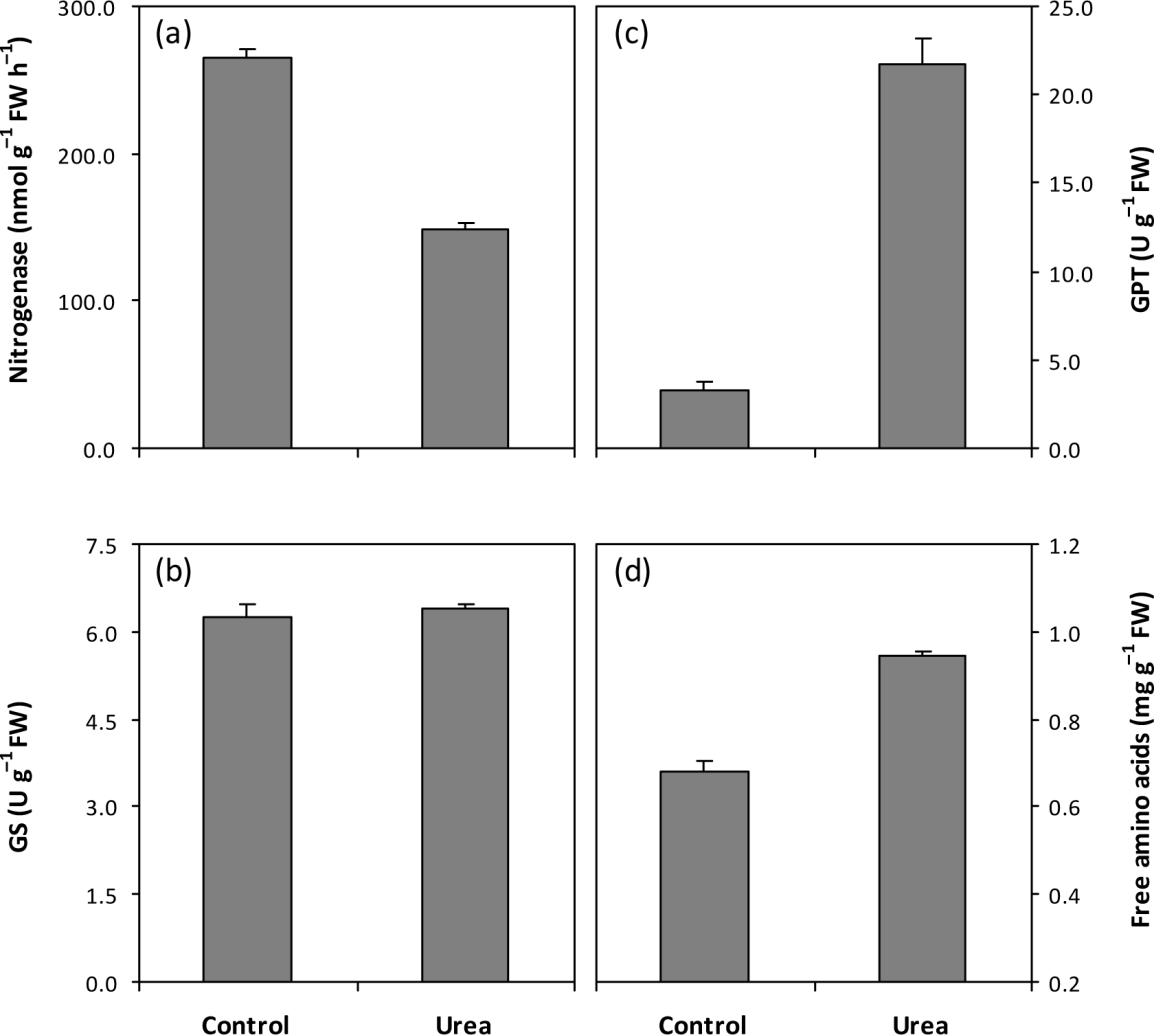
Effect of urea application on activities of nitrogenase, glutamine synthetase (GS) and glutamic-pyruvic transaminase (GPT) and concentration of free amino acids in *Azolla*. Vertical bars represent SE (*n* = 6). The difference between control and urea treatment in (b) is not significant at the 0.05 probability level.

All results described hereafter are presented relative to that in the control. Urea-treated *Azolla* showed a 44% decrease in nitrogenase activity (Fig 2A), which is in agreement with the findings of previous studies [2, 8]. Prior to this study, information on the effect of urea application on N metabolism in *Azolla* was limited. GS is a key enzyme in the assimilation and control of N metabolism; it is the first enzyme involved in the conversion of inorganic to organic N in most organisms [26]. Our study showed no significant difference in GS activity between the urea-treated and control *Azolla* ferns (Fig 2B), indicating that N assimilation is not affected by urea application. However, further investigations are needed to comfirm this speculation, because glutamate dehydrogenase (GDH) is can also perform this function in the aminating direction, especially under high levels of NH_4_ [27]. In addition, 660% increase in GPT activity after urea treatment was observed (Fig 2C). GPT is a key regulatory enzyme that catalyzes amino from glutamic acid to transfer pyruvic acid to form alanine and α-ketoglutaric acid [28]. This enzyme plays an important role in plant adaptation to adverse conditions, including changes in amino acid metabolism to maintain a supply of amino groups to ensure immediate protein synthesis upon cessation of the adverse conditions [29]. Moreover, free amino acids are known to play significant roles in osmotic adjustment in plants subjected to adverse conditions [30]. Increases in the free amino acid pool (11–52%) have been reported in various plants exposed to salt and temperature stress [31, 32]. Similarly, in this study, concentration of free amino acids was 39% higher with urea treatment (Fig 2D). These results also reveal that urea application induced an increase in amino acid metabolism in *Azolla*. Amino acid metabolism is a common process in N assimilation, carbon fixation, and secondary metabolism [33]. Fahnenstich et al. [34] reported that amino acids derived from tricarboxylic acid (TCA) cycle acids increase whereas those from glycolysis intermediates decrease in glycolate oxidase-overexpressing *Arabidopsis*. Ishikawa et al. [35] observed that increased amino acid metabolism depletes glycolysis and TCA cycle intermediates in rice cells under oxidative stress. In this study, we did not determine the effect of urea application on the amino acid composition in *Azolla*. Therefore, further studies are needed to determine the effect of urea application on the physiological functions and regulatory mechanisms governing amino acid metabolism in *Azolla*.

### Effect of urea application on membrane lipid peroxidation in *Azolla*

**Fig 3:**
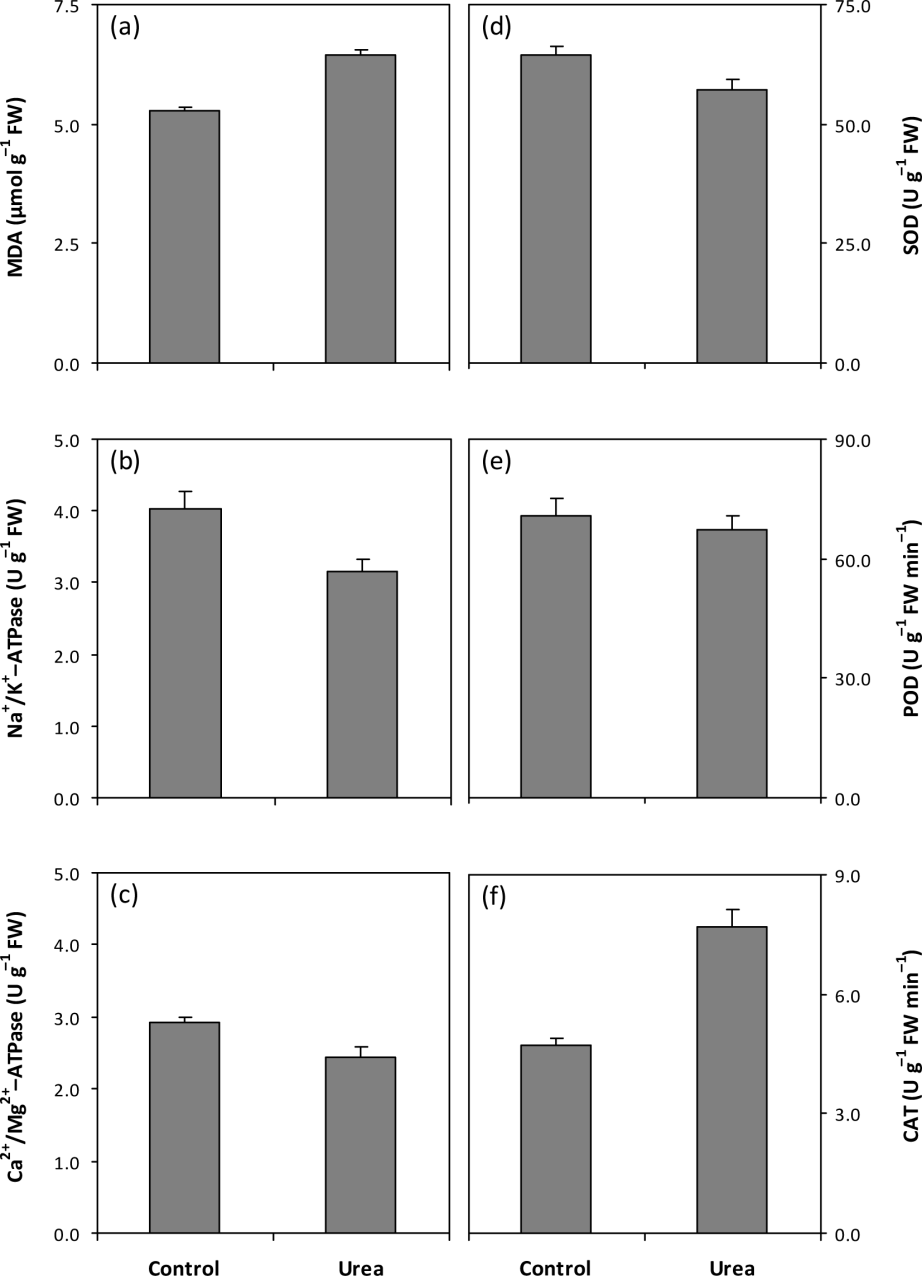
Effect of urea application on concentration of malondialdehyde (MDA) and activities of Na^+^/K^+^-ATPase, Ca^2+^/Mg^2+^-ATPase, superoxide dismutase (SOD), peroxidase (POD) and catalase (CAT) in *Azolla*. Vertical bars represent SE (*n* = 6). The difference between control and urea treatment in (e) is not significant at the 0.05 probability level.

The urea treatment resulted in a 22% increase in MDA concentration in *Azolla* (Fig 3A). Membrane lipid peroxidation can cause further deleterious consequences such as inhibition of ATPase activity [11, 12]. This is supported by the observations in the present study that Na^+^/K^+^-ATPase and Ca^2+^/Mg^2+^-ATPase activity after urea treatment was respectively 21% and 16% lower (Fig 3B and 3C). Although antioxidative enzymes are produced to protect plant cells against oxidative injury [15], free radicals can not be scavenged thoroughly in various plants due to the excessive amount of free radicals generated or the severe weakening of its scavenging capacity [36]. A lower scavenging activity in plants indirectly reflects a decrease in the activity of antioxidative enzymes [37]. In this study, urea treatment resulted in an 11% decrease in SOD activity (Fig 3D). The difference in POD activity was not significant between the urea-treated and control ferns (Fig 3E). *Azolla* subjected to urea treatment showed a 64% higher CAT activity (Fig 3F). These results indicate that the decrease in SOD activity was partially responsible for membrane lipid peroxidation in *Azolla* after urea application. Furthermore, our results also demonstrate that SOD and CAT activities were more sensitive to urea application than POD activity (Fig 3D–3F). These findings suggest that the responses of various antioxidative enzymes to adverse conditions are plant-specific [38–40]. More importantly, the present study showed that the SOD activity response was inversely correlated to that of CAT activity after urea application (Fig 3D and 3F). In plant antioxidative systems, SOD removes O_2_^–^, whereas CAT decomposes H_2_O_2_ [37]. Therefore, the reduction in SOD activity might have occurred prior to the disruption of the H_2_O_2_-scavenging system, and membrane lipid peroxidation in urea-treated *Azolla* was induced mainly by O_2_^–^. However, further investigations should be conducted to confirm these assumptions.

## Conclusions

Urea treatment of *Azolla* induced an increase in GPT and CAT activity and free amino acid and MDA concentrations and a decrease in nitrogenase and SOD activity. These findings indicate that urea application promotes amino acid metabolism and membrane lipid peroxidation in *A*. *pinnata*.
